# Abnormalities of lipid metabolism in the progression and treatment of depression

**DOI:** 10.3389/fpsyt.2025.1589663

**Published:** 2025-05-29

**Authors:** Xinchi Luan, Xuezhe Wang, Ying Shi, Xinyi Zhang, Yilin Wang, Miao Zhou, Zhaoyi Wu, Zijiao Liu, Xiaoxuan Li, Lihua Zhang, Tianyi Shao, Ruolan Chen, Xianming Chu, Hongyun Wei

**Affiliations:** ^1^ Department of Oncology, Key Laboratory of Cancer Molecular and Translational Research, The Affiliated Hospital of Qingdao University, Qingdao, Shandong, China; ^2^ Department of Cardiology, The Affiliated Hospital of Qingdao University, Qingdao, China; ^3^ Department of Gastroenterology, the affiliated hospital of Qingdao university, Qingdao, Shandong, China; ^4^ Center of Tumor Immunology and Cytotherapy, Medical Research Center, The Affiliated Hospital of Qingdao University, Qingdao, Shandong, China; ^5^ School of Basic Medicine, Qingdao University, Qingdao, Shandong, China; ^6^ Shandong First Medical University & Shandong Academy of Medical Sciences, Jinan, China

**Keywords:** depression, lipid metabolism, dyslipidemia, biomarkers, therapeutic targets, comorbidity

## Abstract

Depression represents a global health challenge with complex etiology and significant societal impact. Recent advancements reveal a critical role of lipid metabolism dysregulation in the pathogenesis and severity of depressive disorders. This review elucidates the impact of lipid imbalance on depression highlighting how dyslipidemia not only makes individuals prone to depression but also exacerbates the progression of depression (including the occurrence of some comorbidities of depression). On this basis, we have summarized that abnormal lipid metabolism may serve as a diagnostic and therapeutic target for depression. We discuss the perturbation of lipid homeostasis in depression, marked by altered triglycerides and high-density lipoprotein levels, and the potential of these lipids as biomarkers for diagnostic precision and therapeutic efficacy. Special emphasis is placed on the molecular mechanisms where lipids influence neuronal function and stress responses, thereby affecting mood and cognitive outcomes. Furthermore, we explore the comorbidity of depression with other systemic illnesses, suggesting a shared lipid-mediated metabolic pathway. Given the integral role of lipids in neural architecture and signaling, targeting lipid metabolism could offer novel therapeutic avenues, enhancing neuroplasticity and potentially mitigating depressive symptoms. Our synthesis aims to pave the way for future investigations into lipid-centric strategies to combat depression, advocating for a metabolic reorientation in mental health therapeutics.

## Graphical Abstract

Graphical abstract highlights the role of lipid dysregulation in the pathogenesis of depression, links lipid profile alterations to neuronal dysfunction, stress response, and comorbidities, and proposes diagnosis/treatment targeting lipids. Created using images from Servier Medical Art, licensed under CC BY 4.0; created by Figdraw.com and created using images from Scidraw.io, licensed under CC BY 4.0.

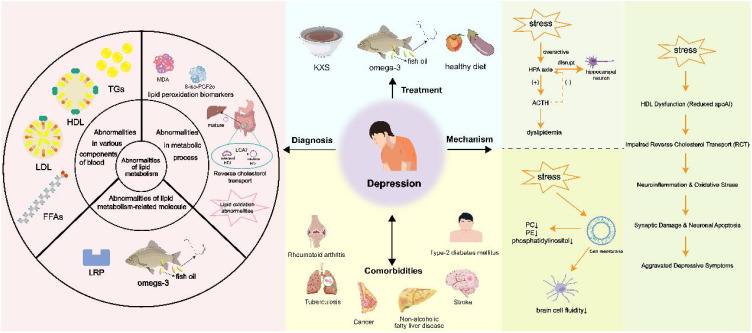

## 1 Introduction

Depression is a widespread psychological disorder that profoundly impacts individuals, families, and society, the World Health Organization (WHO) highlights that depression is the leading cause of disability globally and substantially contributes to the overall disease burden (1, 2). Recent research highlights that 3.8% of the population suffers from depression, including 5% of adults (with 4% being men and 6% women) and 5.7% of individuals aged 60 and older (3). Globally, depression affects over 280 million individuals and plays a crucial role as a risk factor for suicide. Each year, more than 700,000 people die by suicide, making it the fourth leading cause of death among those aged 15 to 29 (2, 3). Its severe consequences have significantly increased suicide rates, highlighting the urgent need for effective management and intervention strategies (4). The multifaceted etiology of depression complicates early diagnosis, often resulting in delayed treatment and worsening symptoms (5). Patients frequently experience recurrent and treatment-resistant episodes, which can undermine the overall efficacy of existing therapies (6). Furthermore, the onset and progression of depression involve intricate regulatory networks, encompassing genetic, biological, psychological, and environmental factors (7). Although some progress has been made in research, the underlying mechanisms of depression remain poorly understood, highlighting the need for continued investigation to uncover its complex pathology and improve therapeutic outcomes.

Lipids, such as Triglycerides (TGs), phospholipids, and cholesterol, are essential for cellular function. They are vital parts of cell membranes, act as energy storage molecules, and function as signaling molecules (8). Lipid metabolism is a core biological process involving the synthesis, degradation, and regulation of lipids in the body. Sustaining cellular homeostasis and overall health relies on the precise regulation of lipid metabolism (9, 10).

Dyslipidemia, marked by atypical blood lipid levels, is increasingly recognized as a significant factor in depression’s pathophysiology. Extensive research has shown that changes in lipid profiles, especially increased TGs and reduced high-density lipoprotein cholesterol (HDL-C), correlate with greater risk and heightened severity of depression (11, 12). The mechanisms through which lipid imbalances influence emotional and cognitive functions are complex. The uniformity of lipids plays a vital role in various interconnected mechanisms, such as mood stabilization, anxiety management, and the prevention of suicidal tendencies. These mechanisms involve serotonin transmission (13–15), the generation of new neurons (neurogenesis) (16, 17), and protection against excitotoxic damage (18). Disruptions in lipid metabolism can affect neuronal function, leading to altered neurotransmission, inflammation, and impaired brain plasticity, all of which are implicated in the development and progression of depressive symptoms (19–21). From a clinical standpoint, abnormal lipid levels appear to be linked to depression, its intensity, and the long-term progression of the condition (22). A noticeable relationship exists between depressive symptoms and fluctuations in blood lipid concentrations, encompassing total cholesterol, low-density lipoprotein (LDL), high-density lipoprotein (HDL), triglycerides, and ω-3 polyunsaturated fatty acids (PUFAs) (23–25). These connections may differ when comparing males and females (26). Additionally, dyslipidemia may promote chronic low-grade inflammation, which has been linked to mood disorders and cognitive decline. Omega-3 fatty acids, known for their anti-inflammatory properties, have been shown to mitigate these effects by improving synaptic plasticity, enhancing neurogenesis, and potentially alleviating depression symptoms (27, 28).

**Figure 1:**
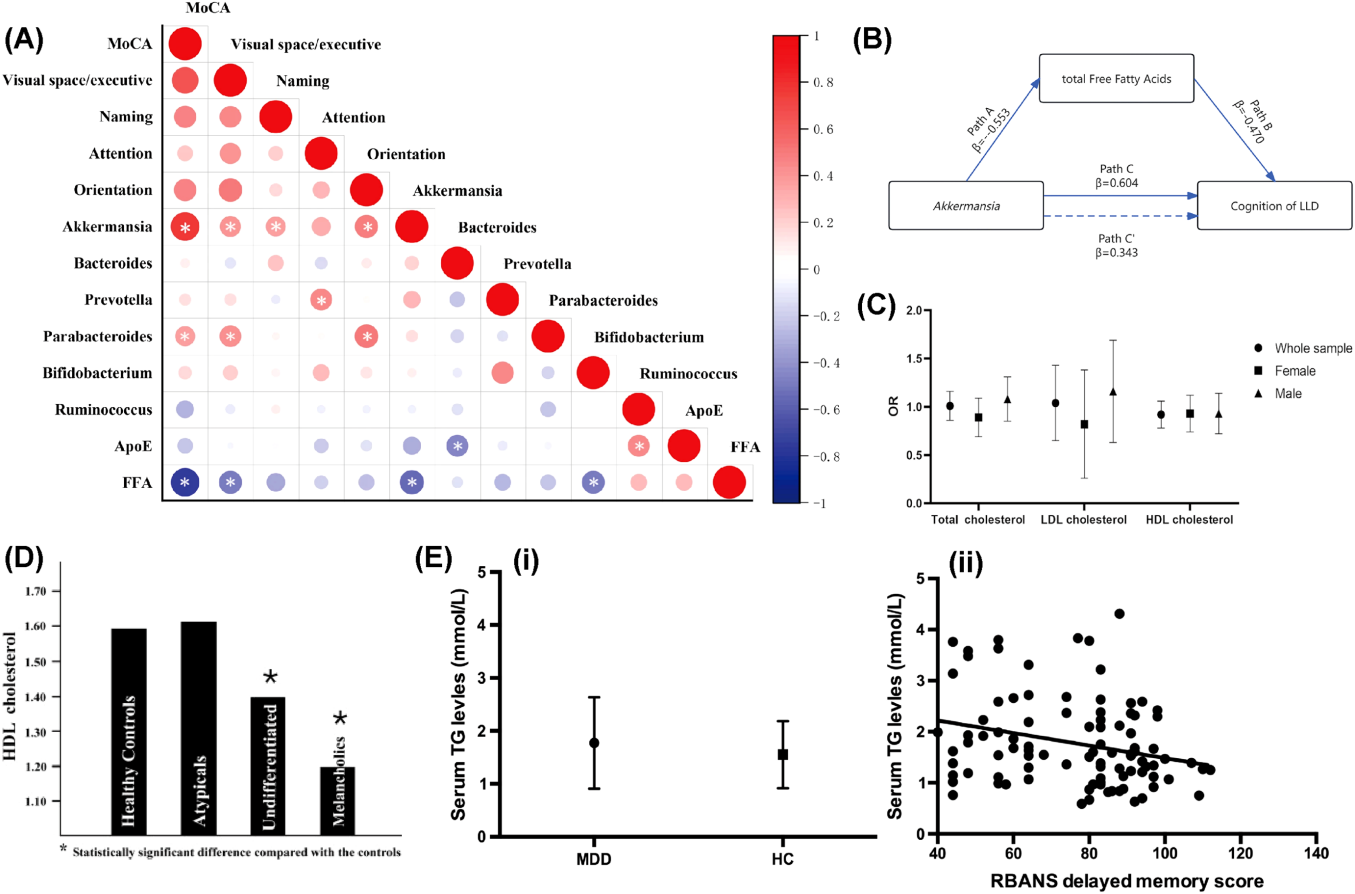
Abnormal lipid metabolism in the process of depression. **(A)** Connections between gut microbiota, lipid metabolites, and cognitive function scores. Reprinted from (170), licensed under CC BY 4.0. **(B)** Mediation model of total FFAs in the link between Akkermansia and cognitive functions. Reprinted from (170), licensed under CC BY 4.0. **(C)** Association between total cholesterol and depression. Reprinted from (58), licensed under CC BY 4.0. **(D)** HDL cholesterol levels in healthy individuals and those with various major depression subtypes. Reprinted from (69) with permission from Elsevier. **(E)** Elevated triglyceride levels have been linked to cognitive decline in people with major depression. (i) Serum TG levels were notably higher in MDD patients compared to healthy controls. (ii) A significant correlation was found between serum TG levels and delayed memory score (r = -0.267, p = 0.008). Reprinted from (97) with permission from Elsevier. Abbreviation: MoCA, Montreal Cognitive Assessment; FFAs, free fatty acids; LLD, Late-Life Depression; ApoE, apolipoprotein E.

Lipid metabolism is crucial for the occurrence and development of many diseases (29). Regarding depression, dysregulation of lipid metabolism has become an important factor in its pathophysiology. Previous studies have shown that when cortisol levels become extremely high and the brain is unable to control the excessive production of cortisol, a decrease in the concentration of brain-derived neurotrophic factor (BDNF) is detected, which affects the development of depression through tyrosine kinase receptor (TrkB) and p75 neurotrophic factor receptor (p75NTR) (30, 31). On the contrary, depression can itself disrupt lipid metabolism, thereby contributing to further disorders in blood lipids. The stress response associated with depression may activate the hypothalamic-pituitary-adrenal (HPA) axis, resulting in elevated cortisol levels. Cortisol inhibits the activities of Δ5- and Δ6-desaturases, reducing the synthesis of ω-3 long-chain polyunsaturated fatty acids (LCPUFAs), such as eicosapentaenoic acid (EPA), docosahexaenoic acid (DHA), and arachidonic acid (AA). This alteration affects the structural properties of fatty acids, consequently influencing lipid synthesis and degradation processes (32, 33). Abnormal fatty acid metabolism caused by hyperactivity of the HPA axis may increase the risk of depression recurrence and cardiovascular diseases by affecting neuromembrane function and inflammatory responses (32). This bidirectional relationship highlights the complex interaction between dyslipidemia and depression, where dyslipidemia exacerbates depressive symptoms, and depression in turn further aggravates lipid imbalance. Meanwhile, gut microbiota may influence lipid metabolism, thereby impacting neuropsychiatric disorders (**Figures 1A, B**). Furthermore, disturbances in lipid metabolism are related to complications of depression and other conditions like cancer, tuberculosis, and cardiac disease (34–36). Recent studies have shown that targeting the lipid metabolism-related gene PTGS2 can inhibit neuronal ferroptosis while promoting immune responses, thereby alleviating breast cancer and associated depression (36). This suggests that there may be a shared metabolic pathway, which warrants further investigation.

During the treatment of depression, alterations in lipid metabolism have also been observed, with various lipid components potentially undergoing alterations that may serve as biomarkers for evaluating treatment efficacy (37). This underscores the possibility of targeting lipid metabolism in managing depression to restore metabolic balance and improve clinical outcomes.

Against the backdrop of depression, emerging evidence has unveiled a bidirectional causal relationship between dyslipidemia and depressive symptoms; however, the precise molecular mechanisms underlying this association remain to be fully elucidated. Therefore, further investigations are needed to uncover these mechanisms and their implications for therapeutic interventions. This review will elucidate the relationship between lipid metabolism and depression, providing a foundation for further investigation in this area.

## 2 The role of lipid metabolism in neurotransmitter systems, neuroplasticity, and depression

Abnormal lipid metabolism influences the function of monoamine neurotransmitter systems through multiple pathways. The functionality of serotonin (5-HT) receptors is closely associated with the cholesterol content of lipid rafts (14), where cholesterol stabilizes the conformation of the 5-HT1A receptor and promotes G-protein coupling, modulating inhibitory signaling in mood-related brain regions. Omega-3 polyunsaturated fatty acids (such as DHA) enhance synaptic membrane fluidity and optimize serotonin transporter (SERT) activity. DHA deficiency may exacerbate depressive phenotypes through neuroinflammation (38). The dopamine system is similarly regulated by lipid dynamics, with sphingolipids (e.g., ceramide) disrupting the integrity of prefrontal cortical lipid rafts and interfering with the localization of dopamine D1 receptors and cAMP signaling pathways (39). Additionally, elevated triglycerides (TG) may inhibit tyrosine hydroxylase activity through oxidative stress, affecting dopamine synthesis (38).

The regulation of neuroplasticity is also closely linked to lipid metabolism. Lipid peroxidation products (such as 4-hydroxynonenal) inhibit the TrkB receptor signaling pathway, impairing the function of brain-derived neurotrophic factor (BDNF) (38). Omega-3 fatty acids upregulate BDNF expression through epigenetic modifications (e.g., histone acetylation) while inhibiting pro-inflammatory cytokines (39). Cholesterol sulfate activates NMDA receptors to enhance dendritic spine formation, with its deficiency in depression being associated with hippocampal atrophy (14).

Gender differences in lipid metabolism significantly impact depression risk. Higher levels of HDL-C in premenopausal women exert neuroprotective effects through anti-inflammatory actions of microglia (40). Estrogen supports synaptic repair by promoting phospholipid synthesis and upregulating APOE expression (39). After menopause, the decline in estrogen, accompanied by a decrease in HDL-C and an increase in LDL-C, exacerbates oxidative stress-related depressive symptoms (40). Testosterone in males regulates hepatic lipase activity to maintain TG homeostasis, and gonadal dysfunction may disrupt this balance, increasing the risk of lipid-driven neuroinflammation (40). Age further complicates the lipid-depression association. In older adults, reduced sphingomyelinase activity leads to an increased ceramide/sphingomyelin ratio, promoting neuronal apoptosis and white matter hyperintensity (39). Omega-3 deficiency during adolescence impairs myelination and prefrontal cortex maturation, increasing susceptibility to stress-induced depression (38). In elderly individuals, elevated very low-density lipoprotein (VLDL) increases blood-brain barrier permeability, facilitating the influx of neurotoxic lipids (39).

Personalized strategies targeting lipid metabolism must consider gender and age differences. Omega-3 supplementation (EPA: DHA ≥ 2:1) is more effective in females, restoring membrane phospholipid balance and modulating the HPA axis (38). Statins (such as atorvastatin) improve depressive symptoms in elderly males by reducing neurotoxic oxysterols (e.g., 27-hydroxycholesterol) (39). PPAR-γ agonists (such as pioglitazone) enhance hippocampal neurogenesis in postmenopausal women through adiponectin-mediated anti-inflammatory pathways (39).

## 3 Abnormal lipid metabolism in the advancement of depression

### 3.1 Abnormalities in various components of blood

#### 3.1.1 Free fatty acids

FFAs are circulating fatty acids in the blood that do not bind to lipoproteins or other molecules but play important roles in energy metabolism, signal transduction, and other processes (41). FFAs, derived from the breakdown of TGs and the phospholipids degradation, are known for their lipotoxic effects. They can traverse the blood-brain barrier either through passive diffusion or via protein-mediated endocytosis, affecting endothelial functions (42).

It is worth noting that P S Mueller et al. observed patients with depression exhibited an average initial FFA concentration of 0.45 ± 0.16 mEq/liter, markedly exceeding the normal value of 0.35 ± 0.12 mEq/liter (P < 0.01) (43). Similarly, X. Yao et al. reported that consumption of a high-fat diet contributes to neurobehavioral abnormalities in adolescents and induces structural changes in the hippocampus. This occurs through the overactivation of microglia, which is associated with increased levels of free fatty acids in the serum (44).

Meanwhile, Emerging evidence points to a lipid-centric pathway in neurodegeneration driven by astrocytic mitochondrial dysfunction (45). Astrocytes, primarily glycolytic, depend on oxidative phosphorylation (OxPhos) for fatty acid (FA) degradation via β-oxidation (45, 46). Disruption of this process, as seen in Astrocyte-Specific Mitochondrial Transcription Factor A Knockout (TfamAKO), causes lipid droplet (LD) accumulation, particularly in the hippocampus and cortex, along with reactive astrogliosis. Excess FA leads to elevated acetyl-CoA, promoting STAT3 acetylation (K685) and phosphorylation (Y705), triggering astrocyte reactivity and proinflammatory cytokine release (45). Lipid-laden astrocytes disrupt homeostasis: neurons increase FA oxidation (FAO), leading to oxidative stress and synaptic dysfunction, while microglia are activated via IL-3 signaling from astrocytes. Lipidomic analysis in TfamAKO mice reveals elevated free FAs, and spatial analysis in 5×FAD mice links LD accumulation to Aβ pathology-rich regions, where astrocytes show impaired FAO and mitochondrial FA trafficking (45). Transcriptomic overlap between TfamAKO and 5×FAD hippocampi indicates shared pathways in lipid dysregulation, neuroinflammation, and synaptic decline (47). This cascade highlights astrocytes as metabolic regulators, with OxPhos deficiency initiating a feedforward loop of lipid toxicity, neuroinflammation, and myelin loss through suppressed FA/cholesterol synthesis. Targeting astrocytic FAO or STAT3 acetylation could mitigate lipid-driven neurodegeneration in depression (48). Collectively, these findings underscore the pivotal role of dysregulated free fatty acids in depression-associated neurodegeneration.

#### 3.1.2 Cholesterol

Cholesterol is a waxy fatty substance generated by the liver that performs several crucial functions in the body, including forming cell membranes, synthesizing vitamin D, and producing hormones (49). Optimal cholesterol levels are essential for receptor functionality, synaptic plasticity, and myelination within the central nervous system (50–54). The link between neuronal dysfunction and depression is well-established (55, 56), and numerous researches have explored the association between cholesterol and neuronal deficits (57). There exists a hypothesis suggesting that the association between depression and reduced cholesterol levels might influence neural functions, positioning cholesterol as a critical element in neurobiology (50). This review will analyze the connections between cholesterol and depression from three angles: low total cholesterol (TC), reduced HDL, and the implications of both low cholesterol and low-density lipoprotein (LDL) (58) (**Figure 1C**).

##### 3.1.2.1 TC

Guey-Mei Jow et al. found that individuals with major depressive disorder (MDD) presented lower TC levels, establishing a negative correlation between Beck Depression Inventory (BDI) scores and serum cholesterol levels. While this might suggest that hypocholesterolemia could partially explain appetite reduction and weight loss frequently observed in depressed patients, the pathophysiological implications remain unclear (59). Mirel LB et al. subsequently suggested a potential link between depressive states and cholesterol dysregulation, but the directionality of this relationship needs to be elucidated (60). Notably, the observed weight loss in depression-associated dyslipidemia may reflect either a metabolic consequence of appetite-related neurovegetative symptoms, malnutrition resulting from depressive behaviors, or shared biological mechanisms influencing both lipid metabolism and mood regulation (61–63).

On the contrary, M Soledad Cepeda et al. performed a large-scale population study and found people with depression had slightly higher levels of TC, higher levels of triglycerides, and lower levels of HDL-C than people with no depression (64). However, these outcomes were specific to the U.S. population, and the limitations suggest that more investigations are needed to validate these outcomes. Furthermore, Qun Zhang and associates utilized logistic regression to assess the link between low cholesterol levels and depression using NHANES cross-sectional data spanning from 2005 to 2018. Their findings, however, did not support the hypothesis of a significant risk association between low cholesterol levels and depression (58).

The relationship between low cholesterol levels and depression remains inconclusive due to conflicting findings reported in the existing literature. While some studies suggest an association between hypocholesterolemia and depressive symptoms, others have failed to establish a consistent or causal link (50–52, 65). Current evidence does not provide strong support for clinically significant concerns that cholesterol-lowering interventions may increase the risk of depression. Nonetheless, further research is warranted to clarify potential subgroup-specific effects and account for confounding factors such as nutritional status, comorbid metabolic disorders, and heterogeneity among depression subtypes. These considerations highlight the need for longitudinal studies with rigorous phenotypic characterization to resolve discrepancies in the current body of research.

##### 3.1.2.2 HDL

HDL is primarily produced by the liver and small intestine. Its main structural protein, apolipoprotein (apo) AI, forms the framework that carries phospholipids and cholesterol in HDL (66). HDL particles in the blood are chiefly responsible for reverse cholesterol transport (RCT), and HDL-C is a crucial component reflecting HDL functionality (67). Emerging evidence suggests that HDL dysfunction extends beyond atherogenicity to neuroprogressive pathways. Mechanistically, HDL exerts anti-inflammatory and antioxidant effects via apoA1 and paraoxonase 1 (PON1), which inhibit lipid peroxidation and neutralize pro-inflammatory cytokines like IL-6 (68). Depletion of HDL-C in depression may impair these protective functions, exacerbating neuroinflammation and oxidative stress—key drivers of synaptic dysfunction and neuronal apoptosis (69, 70).

Clinical studies consistently report lowered HDL-C levels in major depressive disorder (MDD), bipolar disorder (BPD), and schizophrenia, with severity correlating with symptom chronicity and treatment resistance (69, 70). For instance, Soili M Lehto et al. posited a relationship between prolonged depression and reduced levels of HDL-C, which appears independent of lifestyle factors (69) (**Figure 1D**).

This association is further supported by findings in suicidal MDD patients and first-degree relatives of BPD patients, where HDL-C depletion coincides with cognitive deficits and neuroprogression (71). Mechanistic insights reveal that reduced activity of phosphatidylcholine (lecithin)-cholesterol acyltransferase (LCAT) in MDD impairs cholesterol esterification, diminishing HDL maturation and RCT efficiency (72). Concurrently, decreased apoA1 and PON1 levels in depression compromise HDL’s anti-inflammatory capacity, allowing IL-6-driven neuroinflammation to persist (71). Cortisol hyperactivity in MDD may further suppress HDL biogenesis by downregulating hepatic apoA1 expression (73).

HDL dysfunction disrupts lipid raft integrity in neuronal membranes, altering serotonin receptor (5-HT1A) trafficking and monoamine signaling (74, 75). Additionally, oxidized HDL particles enriched in serum amyloid A (SAA) promote blood-brain barrier permeability, facilitating peripheral inflammatory mediators’ entry into the CNS (76, 77). Overall, HDL-C reduction in depression reflects a multifactorial pathology involving LCAT/apoA1/PON1 dysfunction, cortisol-driven lipid dysregulation, and pro-inflammatory HDL remodeling. Targeting HDL functionality—rather than merely its plasma levels—may offer novel therapeutic strategies to mitigate neuroprogression.

##### 3.1.2.3 LDL

VLDL (very low-density lipoprotein), which is predominantly synthesized and secreted by the liver, contains apoB-100 as its primary structural apoprotein. Through sequential metabolic processing in the circulation, VLDL undergoes lipolytic modification to first convert into IDL (intermediate-density lipoprotein) and subsequently matures into LDL (low-density lipoprotein) (78). This metabolic cascade results in LDL inheriting the apoB-100 protein while becoming enriched with cholesterol esters. In contrast to HDL’s reverse cholesterol transport pathway, LDL serves as the principal vehicle for delivering hepatically-derived cholesterol to peripheral tissues (79, 80). This systemic cholesterol distribution is particularly crucial for maintaining membrane integrity in extrahepatic organs, including providing essential support for nervous system function (81, 82). Notably, emerging evidence suggests that LDL-mediated cholesterol transport may play a supplementary role in neuronal membrane maintenance under specific physiological conditions (83). In recent years, LDL-C has been considered a contributing factor for depressive symptoms for aging men (26). Adam Wysokiński et al. found that high LDL-C was linked to depression (84). Contrarily, J. Rabe-Jabłońska and team observed that depressed subjects tended to have lower levels of low-density lipoprotein cholesterol (LDL-C) (85). The variability in results could stem from differences in depression’s severity, demographic factors such as age and gender, and behaviors like suicidality within the study cohort.

In pursuit of definitive conclusions, Claudia Johanna Wagner and her group conducted supplementary studies that corroborated the findings by A. Wysokiński et al., revealing elevated LDL-C levels in depressed individuals (22, 84). Concurrently, Kim et al. proposed that LDL-C levels might influence depression risk, particularly in male adolescents (86). Their research indicated that boys with borderline or high LDL-C levels were more likely to suffer from depression compared to those with normative levels, suggesting a potential association between increased LDL-C and depression risk. Although this study has limitations, LDL-C could be regarded as a routine parameter for evaluating and treating depression. These discoveries establish a platform for further exploration into personalized lipid metabolism-related therapeutic strategies for emotional disorders and for monitoring treatment efficacy.

#### 3.1.3 TG

TGs are the primary circulating lipids in the blood, crucial for adjusting energy equilibrium and stimulating the activity of cholesterol transport proteins (87–89). In a study by Hyo Jung Choi et al., serum apo B was notably positively correlated with overall amyloid-β (Aβ) deposition in the brain. However, multiple linear regression analysis showed that this correlation disappeared when serum TG levels were designated as a control variable, suggesting the apparent relationship between apo B and brain amyloid burden is most likely via its effect on TG (90). Research into the relationship between TG levels and depression has yielded inconsistent findings. For example, Tiao-Lai Huang and colleagues observed significantly elevated TG levels in the blood of individuals suffering from MDD, correlating positively with the severity of depression (91–93). Conversely, U.G.Ö. Ergün et al. reported no substantial differences in TG levels between MDD patients and healthy controls (94). Further, some studies suggested that TG levels in MDD patients were actually lower than those in healthy controls (95).

Therefore, Charilaos Chourpiliadis et al. conducted a large, population-based longitudinal cohort study, establishing a link between increased TG levels and a heightened risk of depression (96). Building on this epidemiological evidence, Tian Nan Shao et al. discovered that augmented TG levels were linked to declined cognitive abilities, particularly in memory and attention (97) (**Figure 1E**), in individuals with major depression. These cognitive impairments may either exacerbate depressive symptoms or arise from shared neurobiological mechanisms, such as disrupted lipid metabolism affecting neuronal function and integrity. Building on these findings, A. H. Behnoush et al. hypothesized that elevated triglyceride (TG) levels could function both as a biomarker for major depression and as a contributor to its pathogenesis, possibly via dual pathways involving metabolic dysfunction and neuroinflammation (98). Collectively, these studies delineate a complex and multifaceted relationship between TG levels and depression: elevated TG levels not only precede the onset of depression but also correlate with its symptomatic severity and underlying pathophysiological mechanisms. These findings underline the necessity of monitoring TG levels in MDD patients and suggest that TGs might serve as viable targets for therapeutic intervention.

In addition, ketone body (KB), a metabolite of TG in the liver, may also influence MDD (99). Under certain metabolic conditions, the liver converts fatty acids into ketone bodies, which are capable of crossing the blood-brain barrier and can serve as an alternative energy source for the brain (100, 101). Recently, accumulating evidence has highlighted alterations in the energy metabolism profile among patients with MDD (38, 102, 103). Notably, elevated levels of beta-hydroxybutyrate (BHB), a common KB, have been observed in studies involving depression-related models (104–106). Some preclinical studies conducted on rats subjected to acute stress revealed a pronounced increase in BHB levels within the prefrontal cortex (PFC) during the acute stress response (107, 108). BDNF has been shown to be an important molecule affecting the onset and progression of depression, and Sleiman et al. identified BHB as a BDNF promoter acting on HDAC2 and HDAC3 to have an effect on depression (31, 109). In a comparative study involving adolescent (n = 11) and adult patients (n = 21) with age-matched healthy controls, both MDD groups demonstrated statistically significant elevations in plasma BHB levels compared to controls (110). While it remains challenging to directly attribute these findings to the pathophysiology of MDD, the results suggest that acute stress responses are associated with increased KB uptake, specifically localized to the PFC.

Collectively, the evidence derived from both observational and mechanistic studies indicates a potential association between elevated triglyceride (TG) levels and depression, as systematically outlined in **Table 1**. However, because of the limitations mentioned above, additional investigations are necessary to confirm these outcomes and explore the underlying mechanisms.

**Table 1:** The diagnostic role of lipid metabolism-related biomarkers in depression.

Biomarkers	Depressive change	Sample	Biological mechanism	Reference
FFAs	Increase	Blood	The microbiome-gut-brain axis	(43)
HDL	Decrease	Blood	Inflammatory response	(70)
HDL-C	Decrease	Blood	Inflammatory response	(70)
LDL	Increase	Blood	Immunore action and serotonergic dysfunction	(85)
LDL-C	Increase	Blood	Immunore action and serotonergic dysfunction	(85, 86)
TG	Increase	Blood	The metabolic and inflammatory processes in the brain	(96–98)
MDA	Increase	Blood	Lipid peroxidation	(115)
8-iso	Increase	Blood	Oxidative damage and inflammation and lipid peroxidation	(114)
IL-6	Increase	Blood	Oxidative damage and inflammation	(114)
BA	Decrease	Blood	The microbiome-gut-brain axis, immune system and homeostasis regulation	(326)
Apo A	Decrease	Blood	Genetic factors or metabolic defects cause periodic cerebral hypoxia	(149)
Omega-3 PUFA	Increase	Blood	Modulating purine metabolic, sinflammatory processes and lipid peroxidation	(158) (159–161)
Fatty acid metabolism	Decrease	Blood	Fatty acid metabolism	(160)
Inosine	Decrease	Blood	Regulates purine metabolic pathways. Regulates the release of neurotransmitters such as glutamate and serotonin, synaptic plasticity, and inflammatory processes	(160)
Tryptophan	Decrease	Blood	The dysfunction of serotonergic system contributes to the development of depression, and the synthesis of serotonin in the CNS is dependent on the utilization of tryptophan—the biochemical precursor of serotonin —from plasma to brain	(160)
Interleukin-1 beta (IL-1β)	Decrease	Cerebrospinal fluid	Nflammatory response	(327)
DHEA	Tryptophan levels were Decreased in adult MDD, but unchanged in child and adolescent MDD	Saliva and plasma	HPA axis	(328)

### 3.2 Abnormalities in metabolic process

#### 3.2.1 Lipid peroxidation

Meta-analyses showed that one characteristic of MDD was an increase in lipid peroxidation levels (111–113). Clinical investigations and animal studies on depression have also identified elevated levels of lipid peroxidation markers such as malondialdehyde (MDA) and 8-isoprostane F2 (8-iso) (114–116). F2-isoprostanes, unique lipid peroxidation byproducts, are stable compounds detectable in various biological fluids and tissues (117, 118). MDA, a derivative of the oxidation of apo B-containing lipoproteins, reflects the rate of peroxide degradation (119, 120). Although commonly used as a biomarker for lipid peroxidation, MDA’s stability is less reliable than that of 8-iso-PGF2α, and it is more susceptible to confounding factors such as dietary antioxidants (117). Consequently, for assessing lipid oxidative damage in human studies, measuring F2-isoprostanes is generally more reliable (117, 118, 121). This is also one of the potential targets to evaluate depression severity and the effects of depression treatment.

At the outset, N. Dimopoulos et al. assessed whether depression was associated with precursors such as 8-iso-PGF2α (114). The research highlighted that serum levels of 8-iso-PGFPGF2α were notably higher in elderly individuals with depression compared to the control group. Although the study provided initial evidence linking depression with 8-iso-PGF2α, it was limited by a small sample size and the presence of comorbidities in some elderly subjects that could skew the results. Additionally, the study did not account for potential lifestyle factors like exercise and alcohol consumption. To further explore this relationship, S. Yager et al. conducted statistical controls for living habits, including body mass index, alcohol consumption, and exercise, to exclude potential influencing effects on these behaviors on the association between depression and lipid oxidation (116). Further analyses, controlling for these variables, demonstrated that 8-iso-PGF2α serum levels were significantly elevated in the depression cohort compared to the control group. However, within the depression group, there was no significant correlation between the severity of symptoms and 8-iso-PGF2α levels, suggesting the presence of a threshold effect rather than a dose-response relationship.

The link between depression and lipid peroxidation is not yet fully understood, but researchers have proposed several possible explanations. One theory posits that clinical depression may be linked to behaviors that contribute to oxidative damage, such as smoking, alcohol intake, and a lack of physical activity (122). However, in the examined sample, these behaviors were not correlated with levels of 8-iso-PGF2α, and adjusting statistically for these variables did not diminish the established association between depression and 8-iso-PGF2α. It is also hypothesized that depression could stimulate the production of reactive oxygen species (ROS), thereby leading to heightened lipid oxidation (123). In addition, depression may indirectly influence oxidative stress (OS) by promoting inflammatory responses (116).

#### 3.2.2 Reverse cholesterol transport

**Figure 2:**
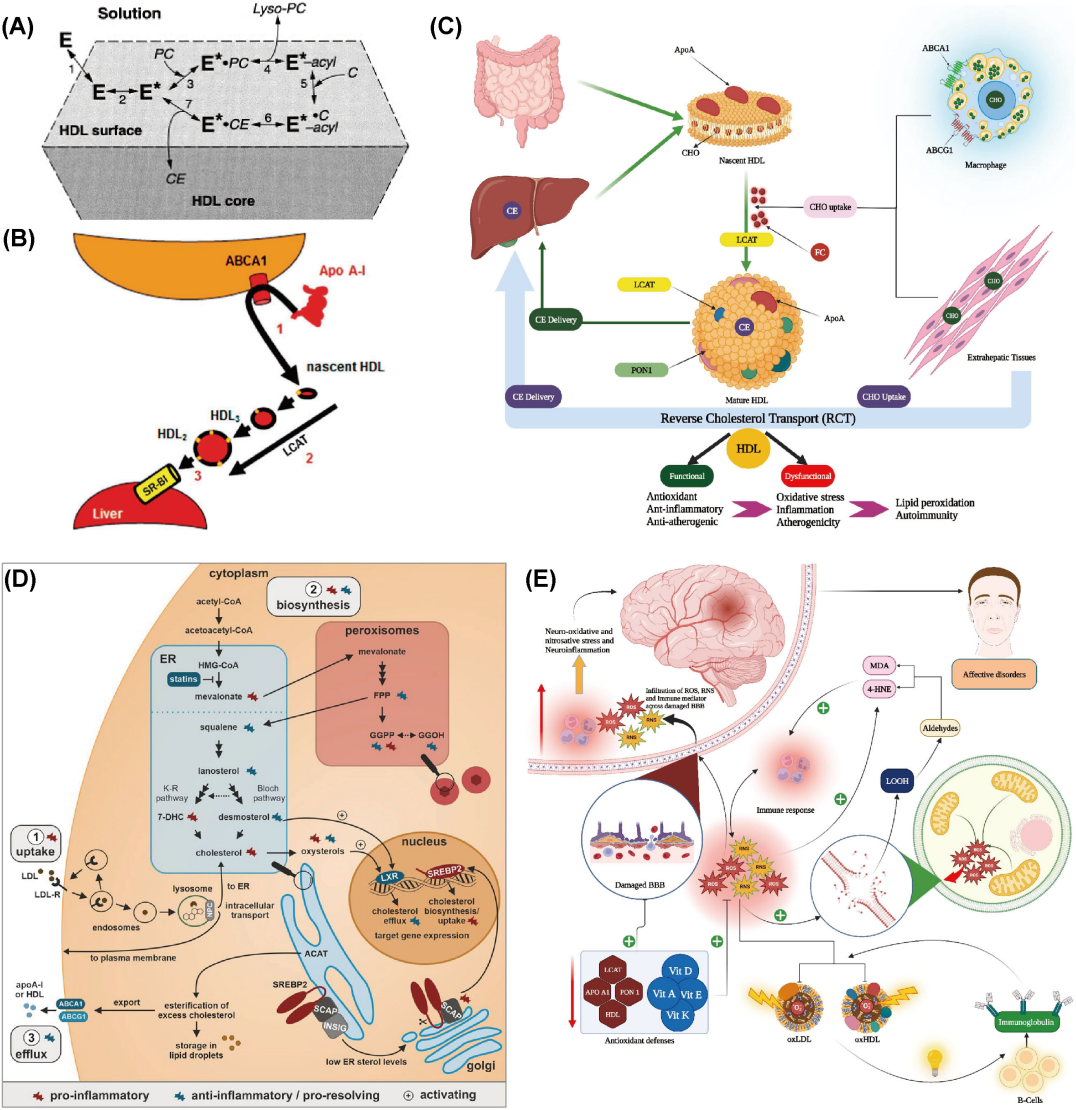
Abnormal metabolic processes during the occurrence of depression. **(A)** Reaction steps of LCAT on HDL surfaces are as follows: E denotes LCAT in solution and bound to HDL lipid surfaces. E* represents the apoA-I-activated LCAT form, while E*-acyl indicates the acylated enzyme. PC and C are LCAT’s lipid substrates, with lyso-PC and CE as the reaction products. Reprinted from (128) with permission from Elsevier. **(B)** The traditional reverse cholesterol transport model involves three steps: (1) efflux of macrophage free cholesterol to apolipoprotein AI through the ATP-binding cassette transporter A1 (ABCA1), resulting in nascent high-density lipoprotein (nHDL); (2) conversion of nHDL to spherical HDL through esterification by phosphatidylcholine (lecithin)-cholesterol acyltransferase (LCAT); and (3) selective hepatic uptake of HDL cholesterol esters via scavenger receptor class B type 1 (SR-B1). Reprinted from (129), licensed under CC BY-NC 4.0. **(C)** Schematic representation of the reverse cholesterol transport (RCT) pathway. CE: Cholesteryl ester, CHO: Cholesterol, FC: Free cholesterol, HDL: High-density lipoprotein, Apo: Apolipoprotein, LCAT: Lecithin–cholesterol acyltransferase, ABC: ATP-binding cassette transporter, PON1: Paraoxonase 1. Reprinted with permission from ref (130). Copyright 2023 Elsevier. **(D)** Cholesterol balance and inflammation regulation. Reprinted from (134), licensed under CC BY-NC 4.0. **(E)** Summary of the impact of reverse cholesterol transport (RCT), reduced lipid antioxidants, and elevated lipid peroxidation on the pathophysiology of major depression and bipolar disorder. Reprinted from (130) with permission from Elsevier.

RCT processes are crucial for moving excess cholesterol from extrahepatic tissues and macrophages back to the liver for metabolic breakdown and excretion (124). HDLs are heterogeneous density lipoproteins with varying size and composition. HDL is important in RCT as it serves as a carrier for cholesterol returning to the liver. Apo AI, produced by the liver and intestines, can aggregate cholesterol and activate cholesterol esterification enzyme LCAT (125–127). LCAT catalyzes the esterification of cholesterol and incorporates cholesterol esters into HDL, thereby aiding in HDL formation and maturation (128) (**Figure 2A**). RCT involves lipid-free Apo AI gathering cholesterol and phospholipids to create nascent HDL particles (127), which then mature into spherical HDL (129) (**Figure 2B**). This process is crucial for cholesterol homeostasis and cardiovascular disease prevention, with LCAT playing a significant role in both RCT and HDL maturation (128, 130)C (**Figure 2C**).

Maes et al. revealed that the ratio of HDL to TC was reduced in individuals with MDD, suggesting that MDD may be associated with impaired RCT function and an elevated risk of atherosclerosis (131). Ketsupar Jirakran et al. reported a reduction in RCT in MDD and suicidal behavior (132). Abbas F. Almulla et al. reached a consistent conclusion that MDD characteristics, including HDL, PON1, Apo A, and LCAT activities, were significantly reduced in RCTs (130). The reduction in RCT may be a pivotal factor in MDD, as it can lead to abnormal cholesterol accumulation in the body, promoting lipid peroxidation and the production of more lipid peroxidation products like MDA. These products have neurotoxic effects and may impair mitochondrial function, leading to declined energy production and increased production of ROS. This can create a self-reinforcing cycle of increased neurotoxicity (71, 133).

The decrease in RCT may be due to an increased inflammatory response, which induces a reduction in the activity or levels of RCT-related molecules such as Apo AI (134) (**Figure 2D**). Alternatively, lipid peroxidation resulting from OS can damage lipids in the cell membrane, thereby impairing normal cholesterol transport. In individuals with depression, levels of antioxidants like vitamin E and coenzyme Q10 are often lower, which may diminish their ability to combat OS and indirectly affect RCT (135). In conclusion, the reduction in RCT may result from multiple depression-related factors, including immune activation, OS, and declined antioxidant defenses. These changes not only affect cardiovascular health but may also exacerbate depressive symptoms and neurodegenerative processes (130) (**Figure 2E**).

#### 3.2.3 Lipid oxidation abnormalities

Furthermore, depression has been associated with the activation of innate immune responses and mild systemic inflammation (136). *In vivo* studies utilizing animal models of inflammation and infection have indicated that systemic lipid oxidation is an integral part of the host response to such conditions (137). As a result, S. Yager et al. proposed that depression might increase oxidized lipid levels by stimulating inflammatory pathways (116). Additionally, individuals with clinical depression may be more prone to behaviors that cause oxidative damage.

Psychological stress is linked to elevated OS in both animal and human models and has been widely reported (138). In addition, depression is often linked to OS (138–140), particularly lipid oxidation (114, 119, 120). Thus, psychological stress may influence the development of depression by triggering abnormal lipid oxidation. Recent investigations have indicated that individuals with advanced depression exhibit a significant imbalance in OS, characterized by elevated levels of free 8-iso-prostaglandins (a lipid peroxidation product) and declined activity of glutathione peroxidase, an important antioxidant defense mechanism (141–143). This imbalance can lead to oxidative-mediated central nervous system (CNS) damage, which is further supported by declined cognitive abilities and more severe depressive symptoms in these individuals. Therefore, interventions aimed at reducing OS may offer potential neuroprotective effects for individuals with depression (144). However, one study reached an opposing conclusion. Black et al. detected no variation in the levels of F2-isoprostane or in the use of antidepressants between the control group and individuals with depression. This large-scale study indicated that there was no increase in OS levels in MDD and anxiety disorders. In addition, the use of antidepressants was related to reduced oxidative DNA damage, indicating that these medications might have antioxidant properties (145).

Overall, there is a complex interaction between depression and abnormal lipid oxidation. Although the exact causal relationship remains incompletely understood, reducing OS and improving antioxidant capacity are both potentially relevant for the prevention and treatment of depression (130) (**Figure 2D**). More researches are needed to further explore the specific mechanisms underlying this relationship in order to offer new strategies for treating depression.

### 3.3 Abnormalities of lipid metabolism-related molecule

#### 3.3.1 Lipid regulatory proteins

LRPs are primarily composed of a variety of apolipoproteins, which are important in the structural stability and metabolic regulation of lipoproteins. They fulfill this function by binding to membrane receptors and regulating enzyme activity. A study by Veni Bharti et al. suggested that certain lipid parameters in individuals with MDD underwent changes, which may be linked to the function of lipid regulatory proteins (146). Sevincock and Sarandol revealed different outcomes in the levels of apo A (one of the subgroups of HDL-C) and apo B (the major protein of LDL-C) in individuals with MDD, with serum apo A levels being lower in individuals with depression (120, 147). What’s more, W E Severus et al. revealed that although there was no dramatic difference in serum apo B levels between individuals with depression and the control group, there were changes in apo B oxidation and serum paraoxonase/arylesterase activity in individuals with MDD, suggesting abnormal lipoprotein metabolism (148).

**Figure 3:**
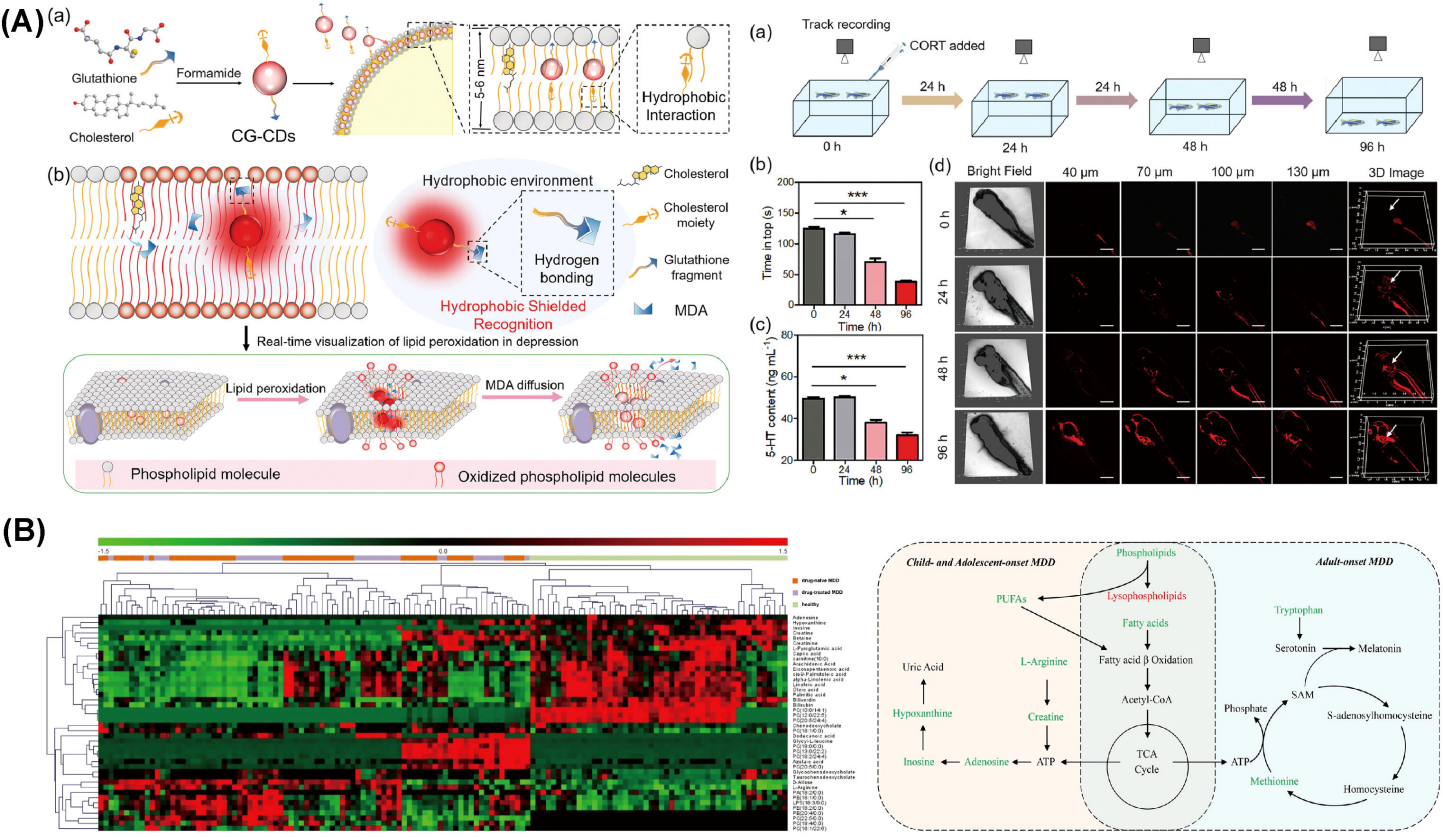
Molecular abnormalities related to lipid metabolism during depression. **(A)** The application of bio-inspired carbon dots developed for real-time monitoring of MDA levels in zebrafish. Reprinted from (143) with permission from John Wiley & Sons. **(B)** Heatmap depicting differential metabolites between depression patients and healthy controls, alongside perturbed metabolic pathways in drug-naïve MDD subjects across children, adolescents, and adults. Metabolites indicated in red are elevated, while those in green are reduced in plasma. Key metabolites include ATP (adenosine triphosphate), fatty acids (such as capric acid, cis-9-palmitoleic acid, dodecanoic acid, oleic acid, palmitic acid, and stearic acid), and PUFAs (polyunsaturated fatty acids), including eicosapentaenoic acid (EPA, ω-3) and arachidonic acid (AA, ω-6). Also represented are SAM (S-adenosylmethionine) and components of the TCA (tricarboxylic acid) cycle. MDD denotes major depressive disorder. Reprinted from (160), licensed under CC BY 4.0.

Due to a lack of evidence and controversial results from earlier studies, Masoumeh Sadeghi et al. designed a study to compare serum apolipoprotein levels between individuals with depression and healthy subjects (149). These results indicated a strong link between serum lipoprotein levels and depression. Regarding lipid profiles in individuals with MDD, findings demonstrated that serum levels of HDL-C and apo A were lower, whereas levels of LDL-C and apo B were higher compared to controls. Given that apo A and apo B are principal components of HDL-C and LDL-C, respectively, these findings align with theoretical expectations (150). Bio-inspired carbon dots, designed by Xiaoyan Wu et al., enable real-time detection of malondialdehyde (MDA), revealing that increased lipid peroxidation, as indicated by elevated MDA levels, is closely associated with lipid metabolism abnormalities and the progression of depression (**Figure 3A**) (143).

These findings demonstrate a significant association between depression and altered serum lipoprotein profiles. In particular, the severity of depression appears to be correlated with increased serum apo B 100 levels and declined serum apo A1 levels (62, 151). Although there are still many limitations, these findings provide new insights for future studies on depression.

#### 3.3.2 Polyunsaturated fatty acid

PUFA, including long-chain omega-3 fatty acids, alpha-linolenic acid (ALA), and omega-6 fatty acids, play a regulatory role in neurotransmitter metabolism and synaptic processes through modulation of synthesis, release, reuptake, and receptor binding, while also play a role in maintaining brain structure and function (152, 153). Veni Bharti et al. Research by Veni Bharti and colleagues has shown that individuals with MDD display reduced PUFA levels (146, 154), and that increasing dietary intake of unsaturated fatty acids (FAs), particularly PUFAs, has been linked to a decreased risk of depression (155–157).

M Maes et al. demonstrated that individuals with MDD had lower omega-3 PUFAs levels in serum phospholipids and cholesteryl esters. Omega-3 PUFAs are essential for brain function and are believed to potentially influence depressive symptoms (158). Similarly, Shoko Tsuchimine et al. demonstrated that levels of omega-3 PUFAs and omega-6 PUFAs were declined in MDD patients (159). Based on these findings, Xinyu Zhou et al. further investigated this issue by conducting a metabolic analysis of plasma samples (160). Compared to healthy controls, individuals with MDD had notably declined levels of EPA and inosine, indicating that omega-3 PUFAs may influence depression through modulation of purine metabolism. Previous reports have linked omega-3 PUFA depletion to inflammation and lipid peroxidation in depression (161). Correspondingly, several studies have indicated that supplementation with omega-3 PUFAs is associated with a lower incidence of depression or mitigation of depressive symptoms in adults (162–164). Further research by Lingsi Zeng et al. proposed that eicosapentaenoic acid (EPA) may exert a therapeutic effect on depression independently, whereas ALA could potentially increase depression risk (165). Additionally, the studies found no link between overall omega-6 PUFA levels and the risk of depression, though Adrenic Acid, a specific omega-6 PUFA, was associated with a reduced depression risk. Similarly, children and adolescents diagnosed with MDD also showed significant disruptions in these metabolic pathways, leading researchers to propose inosine as a potential diagnostic biomarker, as illustrated in **Figure 3B** (160).

Current research highlights a significant association between polyunsaturated fatty acid (PUFA) levels and depression. Observational studies consistently report lower concentrations of omega-3 PUFAs in individuals diagnosed with depressive disorders compared to non-depressed controls. Systematic reviews have corroborated this correlation across numerous epidemiological studies (166). However, the underlying causal mechanisms remain under active investigation. Future research on how to reduce depressive symptoms through modulation of PUFA levels holds promise.

### 3.4 Abnormalities of lipid metabolism of gut microbiota

Recent studies have highlighted the gut-brain axis as a crucial concept in understanding brain function and the pathogenesis of various neuropsychiatric disorders (167) (**Figure 1B**). This axis regulates the gut microbiota and brain function bidirectionally through neural anatomical pathways, neuroimmune and neuroendocrine routes, the intestinal mucosal barrier, microbial metabolites, and the blood-brain barrier. It has been shown that the gut microbiota is closely linked to cognitive function. For instance, Lactobacillus rhamnosus has been demonstrated to regulate cognitive function in anxious-depressed mice (168). Additionally, emerging evidence underscores the gut microbiota as a key regulator of lipid metabolism, particularly in the context of depression.

Studies have shown that chronic social defeat stress (CSDS) in mouse models induces gut microbiota dysbiosis, characterized by an increase in Bacteroidetes and a decrease in Firmicutes and Actinobacteria. This dysbiosis is associated with depressive-like behavior, driven by metabolic reprogramming that includes the accumulation of fatty acids and dysregulated glycerophospholipid metabolism (169). Glycerophospholipids, such as PC, PG, and PI, in the colon are critical in depression, influencing neuronal membrane stability and signal transduction (169).

In elderly patients with depression, a recent cross-sectional study revealed that reduced abundance of Akkermansia in the gut microbiota leads to increased levels of free fatty acids (FFA), contributing to cognitive decline (170) (**Figure 1A**). Mediation analysis further indicated that the impact of Akkermansia on cognitive function in late-life depression (LLD) is mediated by FFA (170). In a depressive-like monkey model, significant changes in the gut microbiota were observed, and lipidomic analysis revealed abnormal lipid metabolism in brain regions (171). Specifically, the prefrontal cortex (PFC) exhibited notable alterations, including a marked increase in 1,2-diacylglycerol (DG) levels. The DG-related pathways in the PFC, such as LPC-PC-PA-DG-TG, were significantly activated, while DG degradation pathways, like DG-PC-LPC, were suppressed (171). These alterations in DG levels and structure disrupt membrane fluidity and signaling pathways, positioning DG as a molecular hub for depressive-like behavior (171). Co-occurrence network analysis further showed that Myoviridae and Prevotellaceae were associated with PFC-DG and negative emotional clustering (171). This suggests that the gut microbiota regulates PFC function through lipid metabolic remodeling, particularly via the DG pathway, providing a new perspective on the gut-brain axis in depression (171).

Additionally, a study by Ke Hu et al. investigated the effects of a high-fat diet combined with stimulation over 16 weeks in ApoE-/- mice. They found that gut microbiota changes, particularly the influence of Desulfovibrio and Akkermansia, along with disturbances in lipid metabolic pathways in the prefrontal cortex and hippocampus, led to the manifestation of atherosclerosis combined with depressive symptoms in the mice (172). This study further supports the involvement of the gut microbiota in atherosclerosis-related depression and reinforces the concept of the “microbiota-gut-brain” axis.

In conclusion, the gut microbiota plays a critical role in lipid metabolism regulation, which significantly influences the pathophysiology of depression. The gut-brain axis, mediated by microbial metabolites and metabolic changes, highlights the importance of microbial diversity in mental health and offers promising therapeutic avenues. Dysbiosis, triggered by chronic stress, alters lipid metabolism—particularly glycerophospholipids like diacylglycerol—and provides a molecular framework for understanding the metabolic disturbances in depression. Moreover, emerging evidence linking gut-derived factors, such as Akkermansia and free fatty acids, to cognitive decline in elderly individuals with depression suggests the gut-brain axis as a potential target for treating depression-related cognitive impairments. Despite these advances, several questions remain. The exact molecular mechanisms through which the gut microbiota influences lipid metabolism and brain function, especially in different depression subtypes, need further investigation. Additionally, translating these findings into clinical therapies, such as microbiota-based treatments or lipid-targeting interventions, poses a significant challenge.

## 4 The role of abnormal lipid metabolism in the diagnosis of depression

Now, MDD diagnosis depends entirely on symptoms and clinical presentation, leading to a high rate of misdiagnosis (173). Furthermore, discovering clinically meaningful diagnostic biomarkers for MDD remains a great challenge. Therefore, research is focused on identifying diagnostic markers for MDD (174, 175).

TIAO-LAI HUANG et al. reported that there were no significant differences in VLDL, LDL, TC, TG, and HDL lipid concentrations, the TC/HDL ratio, or other lipid-related parameters, such as the LDL/HDL ratio, between patients with MDD and normal control subjects. Among treatment-naive MDD patients exhibiting depressive characteristics, males demonstrated higher levels of TG and VLDL, while females exhibited lower levels of HDL. These findings suggest that these lipid profiles may serve as potential biomarkers for distinguishing patients with atypical depressive features (91, 92). Additionally, the results indicate possible sex-based differences in lipid concentrations among individuals with MDD (176). Currently, serum HDL-C and LDL-C levels have been investigated as predictive factors for MDD (43). In addition, Xinyu Liu et al. identified a lipid panel consisting of LPE 20:4, PC 34:1, PI 40:4, SM 39:1,2 and TG 44:2 as possible diagnostic biomarker (93). This combination marker shows good sensitivity and specificity and can distinguish between MDD individuals and healthy controls, making it a strong candidate for lipid-based clinical diagnosis of MDD. In addition, previous studies also showed reduced levels of omega-3 PUFAs and omega-6 PUFAs in individuals with MDD. according to previous studies (159). A deficiency in omega-3 PUFAs is associated with inflammation and lipid peroxidation in depression (161). Thus, omega-3 PUFAs may also serve as potential biomarkers.

It is worth noting that children and adolescents often respond to depression treatment differently from adults (177). One explanation for this difference could be the varying pathophysiological mechanisms of depression between adults and adolescents (178, 179). In pursuit of identifying metabolic changes specific to depression in children and adolescents, it becomes crucial to explore potential biomarkers that reflect these alterations. In this context, Xinyu Zhou et al. undertook an innovative study to discover potential biomarkers for MDD in this demographic by analyzing plasma samples metabolically (160). Their results indicated that, compared to their healthy counterparts, children and adolescents with MDD exhibited significantly reduced levels of capric acid, cis-9-palmitoleic acid, carnitine dodecanoic acid, oleic acid and palmitic acid involved in the fatty acid beta-oxidation pathway (160). Considering these outcomes, investigators suggest that downregulation of fatty acid beta-oxidation could serve as a possible diagnostic criterion for MDD in children and adolescents.

In addition, alterations in lipid metabolism may also involve metabolites related to energy metabolism. Najaf Amin et al. have indicated that gut microbes are crucial in modulating blood lipid levels and that the gut microbiome community is dysregulated in individuals with depression (180). These changes in metabolites not only act as biomarkers for depression but may also offer novel insights into the pathogenesis of the condition. Such findings could enhance the diagnosis of depression and aid in the invention of novel medications.

## 5 The role of lipid metabolism in comorbidities of depression and other diseases

Extensive longitudinal and cross-sectional research has consistently demonstrated a frequent co-occurrence of depression with obesity and metabolic syndrome (181, 182), suggesting shared pathophysiological processes between metabolic disruptions and persistent low-grade “metabolic” inflammation (181). Notably, a cross-sectional study highlighted that individuals with depression, particularly those with anhedonia, showed a higher occurrence of metabolic syndrome (183, 184). Additionally, Luppino FS et al. confirmed the link between depression and obesity, revealing that depression may be a precursor to obesity (185). The metabolic alterations associated with abdominal fat accumulation in obese patients, such as hypertriglyceridemia and declined levels of HDL-C, have been well documented (186). These findings underscore the need for a holistic approach to treating MDD, taking into account the significant metabolic components involved. Such insights could lead to more effective interventions for addressing both mental health and metabolic disturbances.

### 5.1 Tuberculosis

TB is a chronic inflammatory condition caused by Mycobacterium tuberculosis (Mtb), posing a serious health risk (187). Epidemiological studies highlight a high incidence of depression and anxiety among TB patients (188), suggesting bidirectional interactions between mental disorders and TB pathophysiology. Mechanistically, lipid metabolic reprogramming serves as a critical nexus linking depression and TB susceptibility.

During TB infection, Mtb enters alveolar macrophages (MΦs) and induces a “foamy” phenotype characterized by lipid body accumulation (189–191). These lipid droplets are hydrolyzed by bacterial lipolytic enzymes to release fatty acids, which fuel Mtb persistence and cell envelope synthesis (192–194). Notably, host peroxisome proliferator-activated receptor gamma (PPAR-γ), a lipid-activated nuclear receptor, is upregulated in Mtb-infected MΦs via TLR2 signaling, promoting lipid droplet biogenesis and bacterial survival (195–197).

Depression exacerbates this lipid-centric pathophysiology through neuroimmune crosstalk. Depressive states activate microglia and peripheral MΦs via TLR-mediated pathways (198, 199), mirroring Mtb-induced immune responses. Chronic depression alters phospholipid metabolism, reduces serum polyunsaturated fatty acids (PUFAs), and disrupts thyroid hormone-regulated lipid profiles (51, 200–202), creating a lipid milieu favorable for Mtb persistence. For instance, omega-3 PUFA deficiency in depression diminishes anti-inflammatory eicosapentaenoic acid (EPA) and docosahexaenoic acid (DHA), while elevating pro-inflammatory arachidonic acid (AA) metabolites (203–205). In TB, AA-derived eicosanoids like prostaglandin E2 (PGE2) and lipoxin A4 (LXA4) regulate MΦ death modalities: PGE2 promotes apoptosis (limiting bacterial spread), whereas LXA4 induces necrosis (facilitating Mtb dissemination) (206–209). Depression-associated PUFA imbalance may skew eicosanoid production toward LXA4 dominance, exacerbating lung damage through uncontrolled MΦ necrosis (207).

Dietary interventions targeting lipid metabolism represent a therapeutic avenue. Antidepressants like fluoxetine paradoxically induce hepatic lipid accumulation (210), underscoring the need for precision in pharmacological strategies. Omega-3 PUFA supplementation may restore AA/PGE2-LXA4 equilibrium, simultaneously ameliorating depressive symptoms and enhancing TB containment (205, 211).

In summary, depression remodels host lipid metabolism via TLR/PPAR-γ signaling and PUFA dysregulation, fostering an intracellular niche for Mtb survival while impairing anti-mycobacterial immunity. Targeting lipid droplet formation, PUFA metabolism, and eicosanoid balance may break this vicious cycle, offering dual benefits for comorbid depression and TB (212–214).

### 5.2 Cancer

Lipid metabolism reprogramming is a dramatic feature of malignant tumors (215), and tumor growth is intricately related to abnormal lipid metabolism (216, 329). Ferroptosis, a mode of non-apoptotic cell death characterized by high lipid peroxidation, is implicated in cancer development, progression, and treatment (217, 218). Interestingly, disorders in ferroptosis are also linked to the onset of depression (219, 220). Therefore, there is a connection between lipid metabolism and breast cancer-related depression (BCRD). Research has found that quercetin, present in Xiao Yao San, targets the lipid metabolism-associated gene PTGS2, inhibits neuronal ferroptosis, and improves the immune response, thereby alleviating BCRD (36, 221). Moreover, Ye et al. explored the interaction of hepatic epoxide metabolism between depression and breast cancer, highlighting the important role of epoxide metabolism in these comorbidities (222).

Dietary interventions, particularly the intake of omega-3 FAs, are considered potential adjunctive methods for mitigating the effects of comorbid depression and lung cancer, as omega-3 FAs are believed to have mood-regulating benefits (166, 223). Some studies have explored the link between omega-3 addition in cancer patients and psychological symptoms. In a related study, Meihui Zhang et al. observed that dietary interventions enriched with omega-3 FAs could enhance the physical and mental well-being of individuals with squamous cell lung cancer who also suffer from comorbid depression (224). Moreover, the study noted no marked difference in overall serum omega-3 levels between cancer patients with major depression and those without. However, the levels of DHA were significantly elevated in the group with mild depression compared to the other groups (225).

However, some epidemiological studies have reached opposite conclusions (226). Suzuki S et al. found no significant relationship between the total consumption of DHA and EPA and the occurrence of depression in patients recently diagnosed with lung cancer. These findings suggested that DHA and EPA intake did not influence the susceptibility of Japanese cancer patients to depression. Given that Japanese diets typically include higher levels of fish consumption, the antidepressant effects of DHA and EPA may not be as clearly demonstrated in Japanese individuals (226). Moreover, research involving breast cancer patients demonstrated a significant correlation between increased depression scores and higher total blood levels of omega-6 and ALA, yet no correlation with omega-3 levels was observed (227). Findings by Josée Savard et al. similarly did not confirm the efficacy of omega-3 supplements in reducing depression associated with cancer (228).

### 5.3 Stroke

Globally, stroke is the leading cause of disability and presents a significant health challenge (229). Post-stroke depression (PSD) is a common psychiatric issue affecting approximately 33% of stroke survivors, profoundly affecting their daily lives and social interactions (230–232). Investigations have found that individuals with PSD typically had lower levels of HDL-C upon admission (233, 234). However, Shen Huiping et al. demonstrated that the levels and ratios of TG, TC, apo A1, and apo B were not associated with PSD (235, 236). Additionally, the LDL/HDL ratio is considered a useful predictive biomarker for PSD, suggesting that abnormalities or changes in cholesterol transport may be associated with depression (236).

The influence of lipids on PSD could be mediated through multiple mechanisms. Cholesterol, essential for cell membranes and myelin, plays a key role in maintaining plasma membrane fluidity and synaptic functionality (237, 238). Reductions in cholesterol levels may diminish serotonin 5-HT1A receptor binding, disrupt G-protein coupling, and reduce the activity of the 5-hydroxytryptamine transporter (239, 240). Consequently, lower serum cholesterol levels may adversely affect brain lipid concentrations and cell membrane fluidity, impacting serotonergic neurotransmission (241, 242)and potentially contributing to PSD. Wenxia Jiang and collaborators have associated PSD with fecal metabolomic changes, particularly concerning lipid metabolism and dysbalances in gut microbiota (243). Additionally, serum HDL-C levels were distinctly associated with inflammatory markers such as zinc and albumin (244). Low HDL-C levels have been connected to low-grade inflammation, mediated by interleukin-6, which is prevalent in depression (245). HDL-C also correlates with immune markers like the CD4+/CD8+ T-cell ratio (246), indicating a link between lower HDL-C and inflammation in PSD. HDL has antioxidant properties that safeguard LDL from oxidative damage and help eliminate oxidized lipids, thereby inhibiting the generation of intracellular ROS. The levels of HDL-C may reflect the antioxidative activities of HDL (247). OS associated with stroke is fundamental to the pathophysiology of depression (111, 248). Under OS conditions, abnormal alterations in lipids (such as FAs and TGs) can induce neurotoxicity and neurodegeneration, as observed in PSD. Studies found that during acute ischemic stroke, the imbalance between oxidants and antioxidants could further disrupt monoamine reactions by reducing dopamine and serotonin concentrations. This oxidative damage cycle may contribute to PSD (249, 250).

### 5.4 Type 2 diabetes mellitus

Individuals with diabetes have a heightened risk of depression compared to the general population; conversely, depression also significantly increases the risk of developing diabetes (251). Depression not only exacerbates dysglycemia but also increases disability and mortality in diabetic individuals (252). A study conducted in Lithuania revealed significant associations between the emotional states and lipid levels among individuals with T2DM, particularly among females (253). Clinical observations indicated that caution should be exercised when prescribing antidepressants, as misuse could lead to or worsen metabolic dysregulation (254, 255). A Mendelian randomization study from European cohorts confirmed a causal link between TGs, cholesterol levels, and depression phenotypes, indicating that these metabolic factors may bridge the connection between T2DM and depression, potentially mediating their association (256). Further, Wenyu Huang and colleagues identified a causal association linking T2DM with altered lipid profiles, specifically lower HDL-C levels and elevated LDL-C and TG levels (257). Anita NZ et al. found a significant relationship between the severity of depressive symptoms and major depressive episodes in T2DM patients, associated with an oxylipin profile reflecting the degradation of pro-resolving lipid mediators by soluble epoxide hydrolase (258). Additionally, the triglyceride-glucose (TyG) index, a measure of the relationship between fasting plasma TG and glucose levels (259, 260), was independently linked to a higher prevalence of depression among T2DM patients, as reported by Jiaju Ren et al. (261). Notably, supplementation with omega-3 fatty acids, particularly eicosapentaenoic acid (EPA), has been shown to be a safe and effective strategy to reduce depression occurrence in T2DM patients and assist in managing the condition (262).

It is notable that due to the overlap in symptoms between depression and metabolic syndrome (MetS), some researchers have proposed reclassifying depression as “metabolic syndrome type II” (263, 264). Within this framework, PON1, an antioxidant enzyme synthesized by the liver and associated with plasma HDL-C, plays a significant role. PON1 is linked to various conditions including cardiovascular disease (CVD), atherosclerosis, insulin resistance, depression, and T2DM (265–267). Typically, individuals with depression show reduced PON1 and HDL-C levels (70, 268). Similarly, those with diabetes exhibit lower PON1 and HDL-C levels and higher TG levels, correlating with increased oxidized LDL (ox-LDL), atherosclerosis, CVD, and insulin resistance (269). Thus, depression in T2DM may stem from metabolic dysfunctions/MetS alongside diminished PON1 and HDL-C levels.

### 5.5 Non-alcoholic fatty liver disease

NAFLD has emerged as the most common chronic liver condition globally, impacting roughly one-fourth of the population (270). It is intricately associated with metabolic dysfunctions linked to obesity and demonstrates a bidirectional connection with various comorbidities (271, 272). Recent investigations have highlighted a significant correlation between NAFLD and MDD, suggesting that NAFLD might independently elevate the risks of anxiety and depression (273). Conversely, individuals diagnosed with depression are found to be at increased risk for developing NAFLD (274). Additionally, Youssef et al. reported that out of 567 patients with NAFLD, over half exhibited subclinical depression and 14% suffered from clinical depression (275). Analysis of liver biopsy specimens showed that both subclinical and clinical depression correlated with intensified liver cell damage (275, 276). More importantly, depression has been shown to exacerbate liver damage in NAFLD patients (251, 252, 277).

The potential mechanism linking NAFLD and depression may involve alterations in metabolic pathways and inflammatory responses. For instance, studies have demonstrated that chronic unpredictable mild stress (CUMS) in rodent models leads to transcriptional changes in key metabolic enzymes, thereby increasing the synthesis of hepatic fatty acids (278).Chronic stress in murine models has been linked to increased hepatic TGs and cholesterol levels, coupled with a decrease in total visceral fat (279). Su et al. reported that depression aggravates hepatic steatosis in mice and contributes to NAFLD progression by stimulating the HPA axis (280).

Bile acids (BAs) play a crucial role as detergents in lipid digestion and are known to exhibit abnormal metabolic patterns in individuals with NAFLD (281). Beyond their hepatic functions, BAs also exert neuroprotective effects in the brain (282, 283). Notably, cerebral dysfunction has been identified as a potential extrahepatic symptom of NAFLD (284). In CUMS models, significant elevations in serum bile acid levels have been observed, indicating a stress-related dysregulation of BAs (285). Moreover, the role of bile acid receptors such as the Farnesoid X receptor (FXR) and G protein bile acid-activated receptor 1 (GPBAR1) is crucial in regulating inflammatory responses and metabolic disruptions, including lipid imbalances, across both hepatic and neural environments. This highlights the extensive systemic influence of bile acids, affecting a spectrum of physiological and pathological conditions (284).

In experimental settings, mice fed a high-cholesterol diet exhibited signs of hepatic lipid dysregulation and inflammation, indicative of NAFLD, alongside behaviors resembling depression and anxiety. Concurrently, an increase in toll-like receptor 4 (TLR4) expression was observed in both the brain and liver of these animals (286, 287). However, treatment with the insulin receptor sensitizer dicholine succinate effectively countered the elevation of TLR4 and alleviated the anxiety and depressive symptoms induced by the high-cholesterol diet (288, 289).

Antidepressants significantly impact the pathophysiology of NAFLD. Notably, imipramine, by activating the FAM3A-ATP-P2 receptor-CaM-FOXA2-CPT2 pathway, decreases lipid accumulation in hepatocytes, suggesting its potential as a beneficial antidepressant for individuals with concurrent metabolic disorders (290). In contrast, fluoxetine, a widely prescribed selective serotonin reuptake inhibitor (SSRI) for depression, is associated with an increased incidence of metabolic conditions like NAFLD (291, 292). Studies both *in vivo* and *in vitro* have shown that fluoxetine treatment promotes hepatic TG content and lipid accumulation, crucial markers of NAFLD (293, 294). Additionally, fluoxetine impacts prostaglandin production, a process implicated in NAFLD development (295). Specifically, fluoxetine-induced hepatic lipid accumulation involves prostaglandin endoperoxide synthase 1, leading to increased levels of 15-deoxy-Δ^12,14^PGJ2, thereby exacerbating liver damage through complex biochemical pathways (296).

These findings underscore the intricate interplay between NAFLD, cardiovascular metabolic disease risk, and depression, which may be associated with changes in peripheral inflammation and lipid metabolism. Despite the increasing volume of research, there remains a need for further investigation into the precise molecular mechanisms that link these conditions, especially within population-based studies. A more comprehensive understanding of these mechanisms could provide novel approaches and insights for treating and managing NAFLD alongside psychiatric comorbidities.

### 5.6 Rheumatoid arthritis

RA is a chronic inflammatory disorder marked by synovitis and joint destruction, frequently co-occurring with depression, which substantially diminishes the quality of life (QoL) (297). Research indicates that the occurrence of depression among RA patients is two to three times higher than in the general population (298). The interplay between depression and RA progression is well-documented, with a bidirectional causal relationship being a key factor. For instance, worsening RA symptoms can exacerbate depression, and vice versa (299, 300).

Recent research underscores the link between obesity and depression in patients with RA. Studies suggest that RA patients with coexisting depression are more prone to overweight or obesity and exhibit elevated TG levels compared to their non-depressed counterparts (301). Furthermore, active RA is independently linked to decreased HDL-C levels and heightened insulin resistance (301). RA and depression exhibit shared lipid-mediated inflammatory mechanisms driven by dysregulated pro-inflammatory and impaired pro-resolving pathways (200, 302). Both conditions feature elevated prostaglandin E2 (PGE2) and leukotriene B4 (LTB4) via Cyclooxygenase-1 (COX-1)/Cyclooxygenase-2 (COX-2) and 5-lipoxygenase (5-LOX) activation, respectively, which amplify neuroinflammation (microglial activation) and synovial inflammation (fibroblast activation) (200, 302).

Thus, in the context of obesity and hypertriglyceridemia, the association between depression and RA may be more pronounced (186). TG and HDL-C are pivotal in the comorbidity of depression and RA, indicating that therapeutic strategies targeting these metabolic pathways might provide novel avenues for simultaneously managing both conditions.

## 6 The influence of lipid metabolism signaling pathway on depression

Disruptions in lipid metabolism and variations in lipid concentrations can precipitate depression. It has been established that lipid metabolism may be disrupted in individuals subjected to chronic stress or intense noise exposure. These metabolic alterations are governed by the regulation of the HPA axis and changes in membrane fluidity, which may precipitate depressive states.

The HPA axis plays an integral role in the body’s stress response, with cortisol being the primary hormone produced during its activation (32). Chronic stress and intense noise can lead to overstimulation of both the sympathetic nervous system and the HPA axis, enhancing the production of catecholamines, glucocorticoids, and adrenocorticotropic hormone (ACTH) (303, 304). Sustained high cortisol levels can interfere with the negative feedback loop that ordinarily modulates the HPA axis, resulting in its overactivity. This hyperactivity can detrimentally affect the function of hippocampal neurons (305, 306). Imbalances within this signaling pathway may alter neural plasticity, ultimately leading to the development of depression. Additionally, fluctuations in cortisol levels may influence signal transduction processes of membrane-bound receptors due to the hormone’s ability to modify cell membrane fluidity. Such modifications may impede neuronal communication, potentially leading to depressive symptoms. Investigations have also identified a direct correlation between cortisol levels and dyslipidemia (307), further linking altered lipid profiles to depression. Moreover, excessive glucocorticoid activity, induced by chronic HPA axis activation, can foster insulin resistance, increase lipolysis, and raise serum levels of LDL-C, TC, and TGs (308). Changes in these substances can also impact the occurrence of depression. Furthermore, FA metabolism may influence HPA axis activity; studies in individuals with severe depression have indicated that variations in FA unsaturation and chain length can affect the feedback mechanism of the HPA axis (309, 310).

Apart from the HPA axis, cell membrane fluidity, particularly in brain cells, is crucial for the onset of depression. The unsaturation and length of fatty acid chains in membrane lipids critically influence membrane fluidity and cellular membrane functionality (311). Chronic stress may lead to alterations in the composition of membrane phospholipids, characterized by increases in phosphatidylcholine (PC) and phosphatidylethanolamine (PE), and a decrease in phosphatidylinositol levels (312). Such shifts can impair the fluidity of brain cell membranes, potentially contributing to mental health disorders, including depression. Notably, elevated levels of PC have been observed in the prefrontal cortex of patients with bipolar disorder. Furthermore, FAs, vital components of neuronal membranes, are essential for maintaining the structural integrity of brain cells (311, 313). The peroxidation of membrane FAs affects membrane sensitivity to OS (314). Consequently, disruptions in phospholipid or FA metabolism may promote mental illnesses.

In summary, abnormalities in lipid metabolism of glucocorticoids within the HPA axis and membrane lipids can trigger the onset of depression. This suggests that these dysregulated components could be explored as potential therapeutic targets for depression.

## 7 Prospect of lipid metabolism as a therapeutic target for depression

This connection between lipid metabolic disorders and depression indicates that targeting lipid metabolism might offer a viable therapeutic strategy for depression. We have reviewed several methods for treating depression by targeting lipid metabolism and its content.

Firstly, as previously mentioned, excessive activation of the HPA axis can lead to elevated glucocorticoid levels, which can easily trigger depression. Glucocorticoid antagonists can reduce these levels, indicating that controlling the HPA axis can improve depression (315). Among these approaches, Kai Xin San (KXS), which consists of ginseng, hawthorn, polygala, and astragalus (316, 317), has been shown to regulate the HPA axis (318), and thus has potential as a treatment for depression. Additionally, signaling pathways related to lipid metabolism in the peripheral blood of depression patients include HDL-mediated lipid transport, lipid digestion, mobilization, and metabolism. Experimental results demonstrate that KXS significantly modulates these lipid metabolism pathways and exerts an antidepressant effect by adjusting these signaling pathways (318).

Moreover, omega-3 polyunsaturated fatty acids (n-3 PUFAs), particularly EPA and DHA, are proposed to ameliorate or prevent various mental and neurodegenerative disorders by modulating neuroimmune and apoptotic pathways. As integral components of cell membranes, n-3 PUFAs are increasingly recognized for their role in regulating membrane fluidity, inflammatory responses, and antioxidant activity, all of which contribute to neuroprotection (315). The bioactive lipid metabolites derived from EPA and DHA are highlighted as molecular targets that support human hippocampal neurogenesis and mitigate depression. In their research, Song, C., et al. found that EPA alone, or in a higher dosage combined with DHA, was particularly effective in managing MDD (319). Additionally, an imbalance in the w-6:w-3 ratios is associated with maternal postpartum depression (PPD), suggesting that addressing the competition between w-3 and w-6 could be a therapeutic approach for treating depression (320). Furthermore, the accumulation of Aβ is a hallmark of Alzheimer’s disease (AD) and MDD. Reducing Aβ aggregation can improve the progression of AD. Microglia, the brain’s macrophages, continuously monitor their microenvironment through movement and the phagocytosis of Aβ (321). Therefore, microglial activation is essential in the pathogenesis of brain disorders like MDD and AD. Both EPA and DHA can enhance the phagocytic function of microglia, thereby accelerating the elimination of Aβ deposits and exerting neurotrophic and antidepressant effects (322).

In addition, investigations have revealed that fried foods contribute to the risk of mental health conditions in children (323). A recent cross-sectional study found that acrylamide-hemoglobin adducts in fried foods are related to increased depression rates among American adults (324). Additionally, prolonged exposure to acrylamide has been linked to disruptions in brain lipid metabolism and neuroinflammation, notably impacting myelin and phospholipid metabolism (325). Therefore, reducing the intake of fried foods to lower acrylamide exposure may help prevent or treat depression.

In summary, our investigation has reviewed several therapeutic targets for depression, such as n-3 PUFAs. These findings indicate a strong correlation between lipid metabolism and depression, further indicating that targeting lipid metabolism can be a viable approach for preventing and treating depression.

## 8 Conclusion

In conclusion, the intricate interplay between lipid metabolism and depression underscores the significant impact of this field on clinical outcomes. The interplay between dyslipidemia and depressive symptoms highlights the need for a multidisciplinary treatment approach that integrates both metabolic and mental health issues. Given the intricate role of lipid metabolism in neural function and emotion regulation, further research should elucidate its molecular mechanisms. A deeper understanding of these mechanisms will aid in developing innovative therapeutic strategies, potentially revolutionizing the management of depression and enhancing the quality of life for affected individuals.
